# Evaluating the Effectiveness of an Online Targeted Education Programme on Vascular Surgery Aimed at Early Postgraduate Doctors

**DOI:** 10.7759/cureus.70579

**Published:** 2024-09-30

**Authors:** Siddhant A Pherwani, Saad Ahmed, Aditya Gangal, Akash Doshi

**Affiliations:** 1 Urology, Princess Royal University Hospital, King's College NHS Foundation Trust, London, GBR; 2 General Practice, Bute House Medical Centre, Luton, GBR; 3 Internal Medicine, Northwick Park Hospital, London North West University Healthcare Trust, London, GBR; 4 Diabetes and Endocrinology, Royal London Hospital, Barts Health NHS Trust, London, GBR

**Keywords:** foundation doctor, medical education, online education, surgical education, vascular surgery

## Abstract

Introduction

Mind The Bleep (MTB) is a junior doctor-led online education organisation that produces free open-access medical education (FOAMed) resources. This paper describes MTB vascular surgery online education programme. The target audience was early postgraduate doctors completing a rotation in vascular surgery. The aims were to increase participant confidence in basic vascular surgery topics and better prepare them for their role in vascular surgery.

Methods

Speakers included foundation doctors, vascular speciality registrars, consultant vascular surgeons, and a vascular nurse specialist. Post-webinar feedback surveys presented as a five-point Likert scale were used to assess the effectiveness of the course. Wilcoxon signed-rank tests were used to analyse the data.

Results

Over three months, from March to June 2022, nine one-hour targeted education sessions were conducted, with a total of 564 participants. Post-session feedback was obtained from 364 participants (64.6%). There was a significant increase in the participants’ level of confidence in the topic seen across all sessions (p <0.05). The participants rated webinars highly in the domains of engagement (mean 4.31), helpfulness of the content (mean 4.50), and interest (mean 4.39). From the cohort, 200 participants provided feedback on whether attending the webinar better prepared them for a vascular surgery rotation, with 66.5% (n = 133) reporting yes.

Conclusion

The MTB vascular surgery online education program successfully increased participants’ confidence and their preparedness for a role in vascular surgery. As a result, it is a useful adjunct to undergraduate surgical education.

## Introduction

Online targeted education programmes are part of the growing body of educational resources commonly described as 'free open-access medical education (FOAMed),' which include webinars, YouTube videos, websites, question banks, blogs, and podcasts that are disseminated via social media [1]. These FOAMed resources have become ubiquitous for undergraduate and postgraduate students as an adjunct to the traditional pillars of medical education. Concerns regarding FOAMed relate to fact-checking and the accuracy of the information disseminated [2]. Furthermore, access to an online learning environment is not equitable across students, with some having scarce access to equipment, including computers or Wi-Fi. There is a loss of face-to-face interaction and interactivity and a potential for an increased transactional distance between the teacher and learners [3].

The most striking advantage to the use of FOAMed resources is the instantaneous access to medical education, unhindered by the barriers of time and geographical location. Recently, there have been several articles in the literature describing the shift to online platforms in medical education as a result of the COVID-19 pandemic with promising results [4-7]. Furthermore, there have been studies published directly comparing online vs. offline teaching initiatives. A 2022 systematic review and meta-analysis by Gao et. al. investigated online learning vs. offline learning for medical students [8]. The authors included 27 studies, with 21 randomised control trials within the analysis. The analysis reported significantly higher post-test scores and pre- to post-test improvement in the online groups compared to offline. The meta-analysis was limited by heterogeneity in the learning methods used and topics taught between the studies. 

The use of the online platform specifically for education on surgical specialties is less well documented. This is of high importance as there have been a number of publications in the literature highlighting the insufficiency of undergraduate surgical teaching in the United Kingdom (UK) [9-11]. This is of particular concern to vascular surgery, where a growing burden of atherosclerotic disease has resulted in a predicted rise in the demand for vascular surgeons by 67% by 2029 [12]. A 2021 literature review investigated the exposure to vascular surgery in the medical school curriculum [13]. The authors identified that across UK medical students, 25% reported no teaching or exposure to vascular surgery. Of the remaining cohort, the median number of clinical days in vascular surgery was two, and the median number of hours of vascular surgery face-to-face teaching was one. The authors concluded the main deterrent to embarking on a career in vascular surgery was the limited exposure. In the recommendations section, the authors highlighted the role of FOAMed resources in providing exposure to vascular surgery.

In this paper, we describe a FOAMed resource using webinars on vascular surgery, The Mind The Bleep (MTB) vascular surgery online education programme which is targeted at doctors in the UK in their first two years of training, known as the Foundation Programme. This paper aims to describe this FOAMed resource, reflect on its strengths and weaknesses, and how this relates to the wider literature on the subject.

## Materials and methods

Development of the online education programme

We developed this online education programme, through our roles in the medical education organisation, MTB [14]. Mind The Bleep is an open-access medical education website run by UK-based junior doctors. The overall aim of MTB is to provide high-quality, free educational resources to healthcare professionals, including medical students, junior doctors, and allied healthcare professionals.

The programme was aimed at doctors in the Foundation Programme. There were a number of reasons for this. Firstly, the majority of the MTB users are individuals within the Foundation Programme. Secondly, the Foundation Programme curriculum is broad and not specialty-specific. There are no set learning objectives for foundation doctors in vascular surgery. There were also no available online resources for foundation doctors in vascular surgery, and as a result, we felt our programme could address this gap.

We were unable to identify a formal vascular surgery curriculum for medical schools or doctors in the Foundation Programme to use as a framework for deciding on topics covered. We used another online resource, PassMedicine, a UK-based online question bank aimed at undergraduate medical students, to identify vascular surgery topics relevant to medical school final examinations that, as a result, we believed would be important to doctors in their Foundation Programme [15]. We obtained feedback on these choices from consultant vascular surgeons who led a number of our sessions.

Speakers were recruited through email requests. We aimed to include a wide range of speakers, as well as teaching a number of sessions ourselves. Sessions were advertised through the MTB social media pages, including Facebook (Meta Platforms, Menlo Park, CA) and X (formerly known as Twitter, X Corp., San Francisco, CA). The events were shared on the UK Foundation Doctors Facebook groups for all the geographical deaneries. 

The overall aims of our online education programme were: 1. To provide an educational resource on a number of basic vascular surgery topics relevant to junior doctors; 2. To increase the confidence among participants in basic vascular surgery topics; 3. To better prepare medical students and junior doctors for a rotation in vascular surgery

Our targeted education sessions were run on the platform MedAll (MedAll Healthcare, Barking, England), which allows users to create live webinar ‘events’ [16]. Those attending had to sign up for MedAll via their university or institution email to verify their status as a healthcare professional or medical student. Each session lasted up to one hour. Participants were able to interact with the speaker of the session using a discussion box function in the live event. Sessions were conducted on weekday evenings at seven p.m.

Feedback

Feedback forms were disseminated to all participants at the end of each session via email. Five questions collected quantitative data using a five-point Likert scale, with one being the lowest rating and five the highest. These were: 1. Confidence level in the topic before the session; 2. Confidence level after the session; 3. Engagement level in regard to the speaker and the content of the session; 4. Helpfulness of the content in regard to their understanding; 5. Interest in regard to their interest in the content and the format of the session.

Four questions collected qualitative data. These were: 1. Did you feel this session better prepared you for a job as a junior doctor in vascular surgery? 2. What could have been better? 3. What went well? 4. What experience of vascular surgery have you had in your medical training thus far?

Statistical analysis

Data from feedback questionnaires were organised in Microsoft Excel spreadsheets (Microsoft Corp., Redmond, WA) and analysed using KNIME analytics platform software (KNIME AG, Zurich, Switzerland). Wilcoxon-signed rank tests were used to assess for statistically significant differences between pre-and post-session levels of confidence. A p-value <0.05 was deemed statistically significant. Categorical variables are expressed as numbers with a percentage.

## Results

During a three-month period, from March to June 2022, we conducted nine targeted online education sessions with a total of 563 participants. The mean attendance per webinar was 62.5 participants, with a range of 20-96. Feedback was obtained from 346 of the total number of participants (64.7%). There were seven speakers across the nine different webinars (Table 1).

**Table 1:** Summary of the webinars, professional level of speakers, and the number of participants

Webinar title	Speaker	Participants (n)	Feedback (n/% of those attended)
Peripheral Arterial Disease	General surgical registrar	96	79 (82.3%)
Venous Disease	Foundation doctor	74	47 (63.5%)
The Diabetic Foot	Consultant vascular surgeon	70	40 (57.1%)
Carotid Artery Disease	Foundation doctor	75	41 (54.7%)
Aortic Disease	Vascular durgical registrar	70	33 (47.1%)
How to Survive as a Vascular Junior: Part 1 (Management of Vascular Emergencies)	Foundation doctor	55	33 (60.0%)
Wound Management	Vascular nurse specialist	69	34 (49.3%)
Thoracic Outlet Syndrome	Consultant vascular surgeon	34	25 (73.5%)
How to Survive as a Vascular Junior: Part 2 (Pre-operative Assessment in Vascular Patients, Common Co-morbidities in Vascular Patients and Post-operative Complications in Vascular Patients)	Foundation doctor	20	14 (70.0%)

Quantitive feedback

Level of Confidence

Two measures of participant-rated level of confidence were taken, one pre- and one post session. Overall, the mean pre- and post-webinar confidence levels were 2.82 and 4.03, respectively. Across all webinars, we identified a statistically significant increase in participant confidence level (Z = -14.31, p <0.05). The statistical analysis, including Wilcoxon signed-rank tests performed for each webinar, is included in Table 2. 

**Table 2:** Summary of pre- and post-session confidence levels and Wilcoxon signed-rank test results for each webinar

Webinar title	Mean confidence: pre-session	Mean confidence: post session	Z score	p-value
Peripheral Arterial Disease	2.92	4.25	-4.62	0.000001889341
Venous Disease	3.06	4.11	-5.08	0.000000190864
The Diabetic Foot	2.86	3.88	-2.71	0.003358322751
Carotid Artery Disease	2.93	4.10	-6.96	0.000000000002
Aortic Disease	2.52	3.91	-4.30	0.000008395735
How to Survive as a Vascular Junior: Part 1	2.76	3.97	-4.90	0.000000483272
Wound Management	2.68	3.85	-4.00	0.000031813159
Thoracic Outlet Syndrome	2.68	3.88	-4.99	0.000000305380
How to Survive as a Vascular Junior: Part 2	2.86	3.86	-4.49	0.000003601481

Engagement, Interest, and Helpfulness

**Figure 1:**
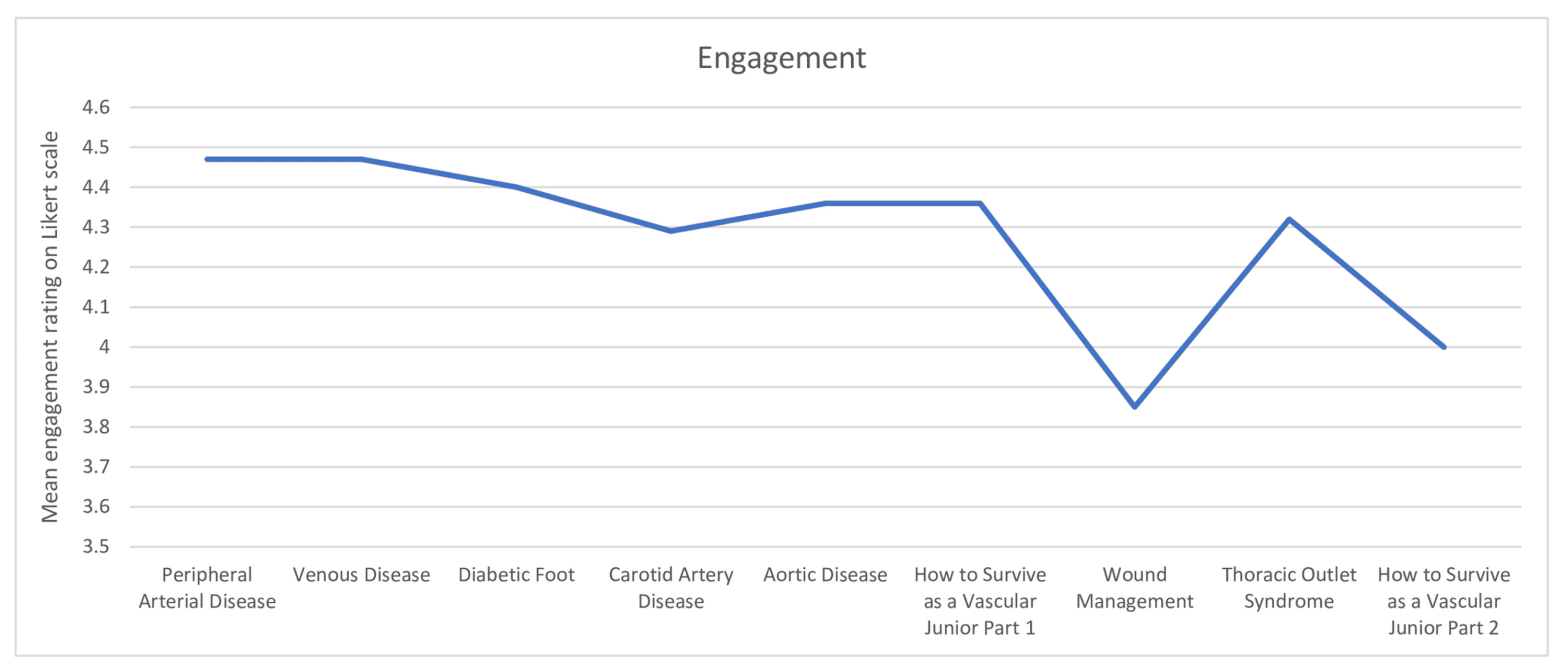
Mean engagement ratings on the Likert scale across webinars

**Figure 2:**
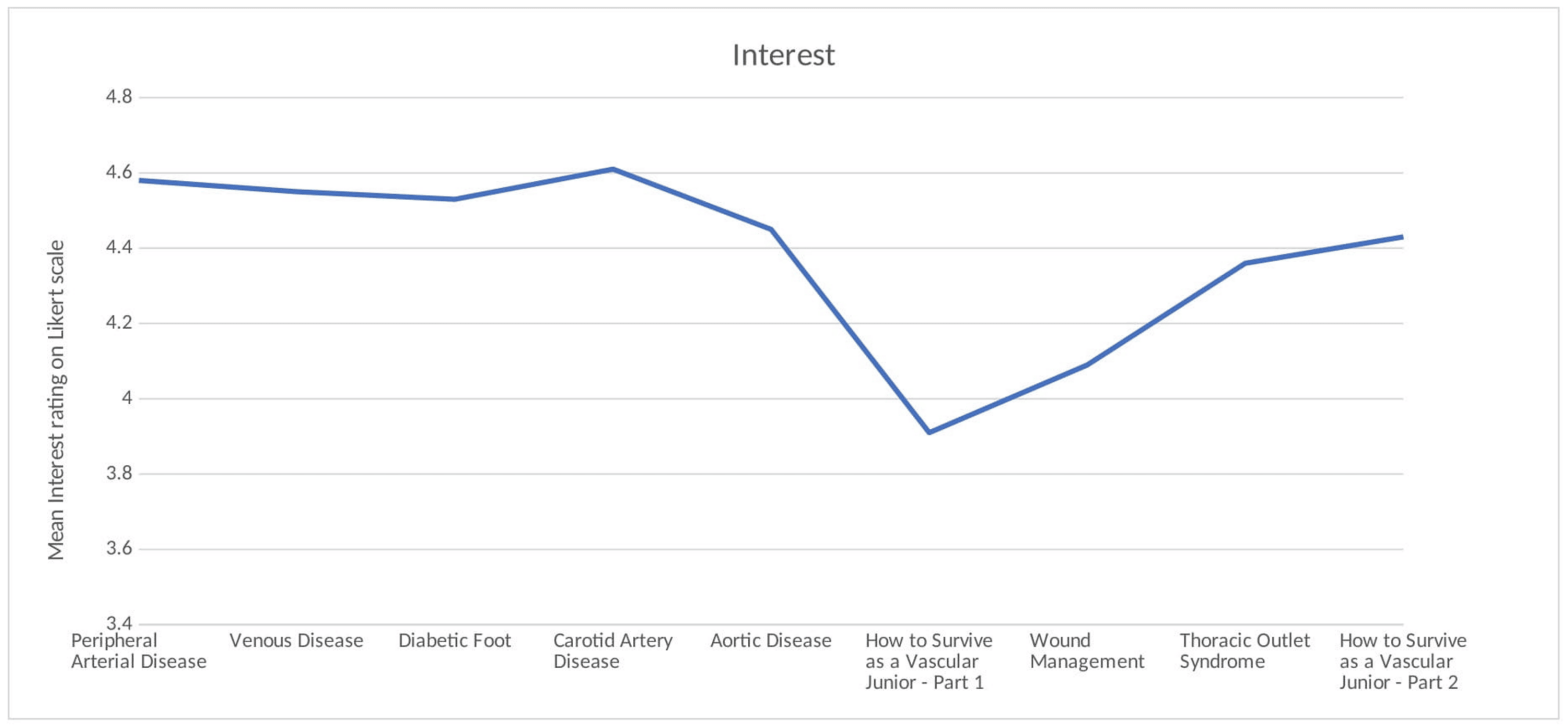
Mean interest ratings on the Likert scale across webinars

**Figure 3:**
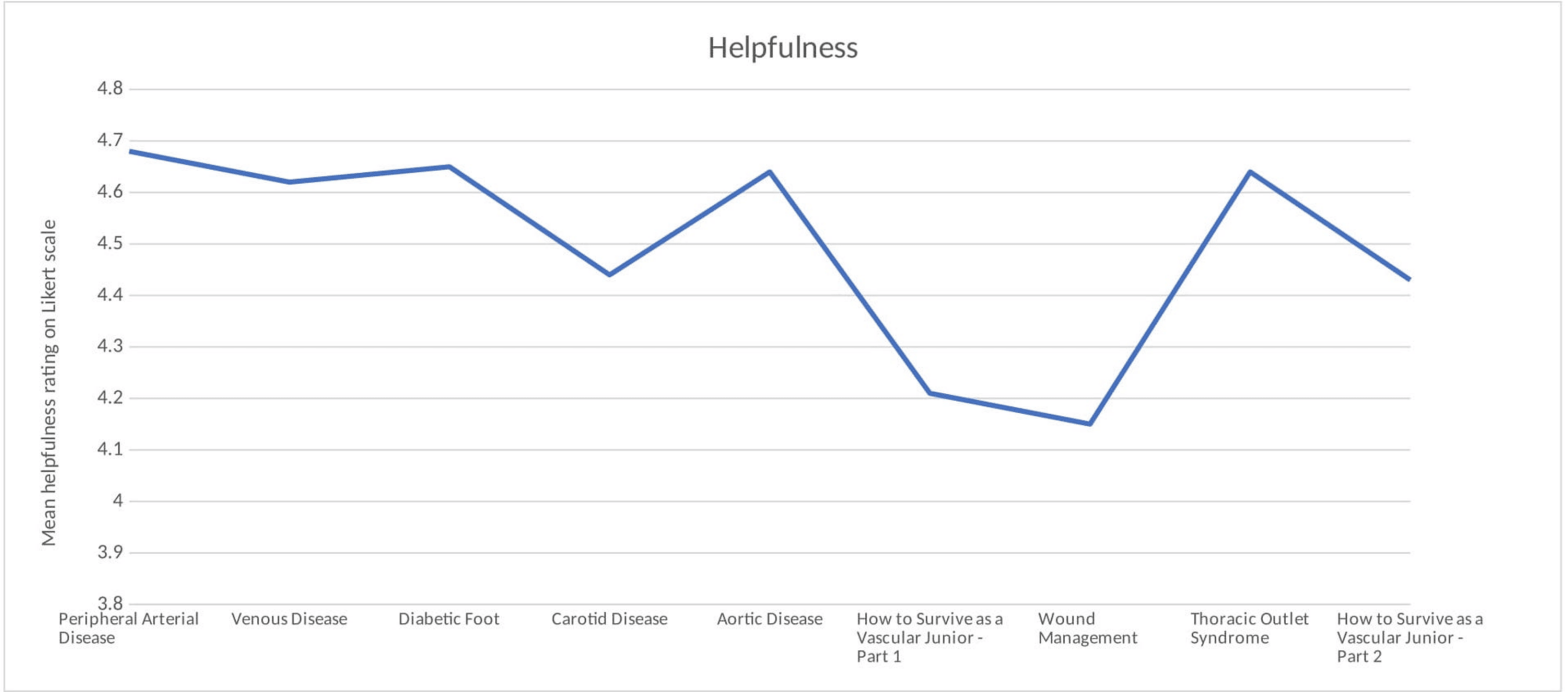
Mean helpfulness ratings on the Likert scale across webinars

The overall engagement rating mean across the webinars was 4.31 on the Likert scale. Of the nine webinars, eight had engagement ratings of greater than or equal to four (Figure 1). The overall interest rating mean across the webinars was 4.39 on the Likert scale. Of the nine webinars, eight had interest ratings greater than or equal to three (Figure 2). The overall helpfulness of the content mean across the webinars was 4.50 on the Likert scale. All nine webinars had a mean helpfulness score of greater than or equal to four (Figure 3).

Qualitative feedback

Do You Feel This Webinar Has Better Prepared You for a Job as a Junior Doctor in Vascular Surgery?

**Figure 4:**
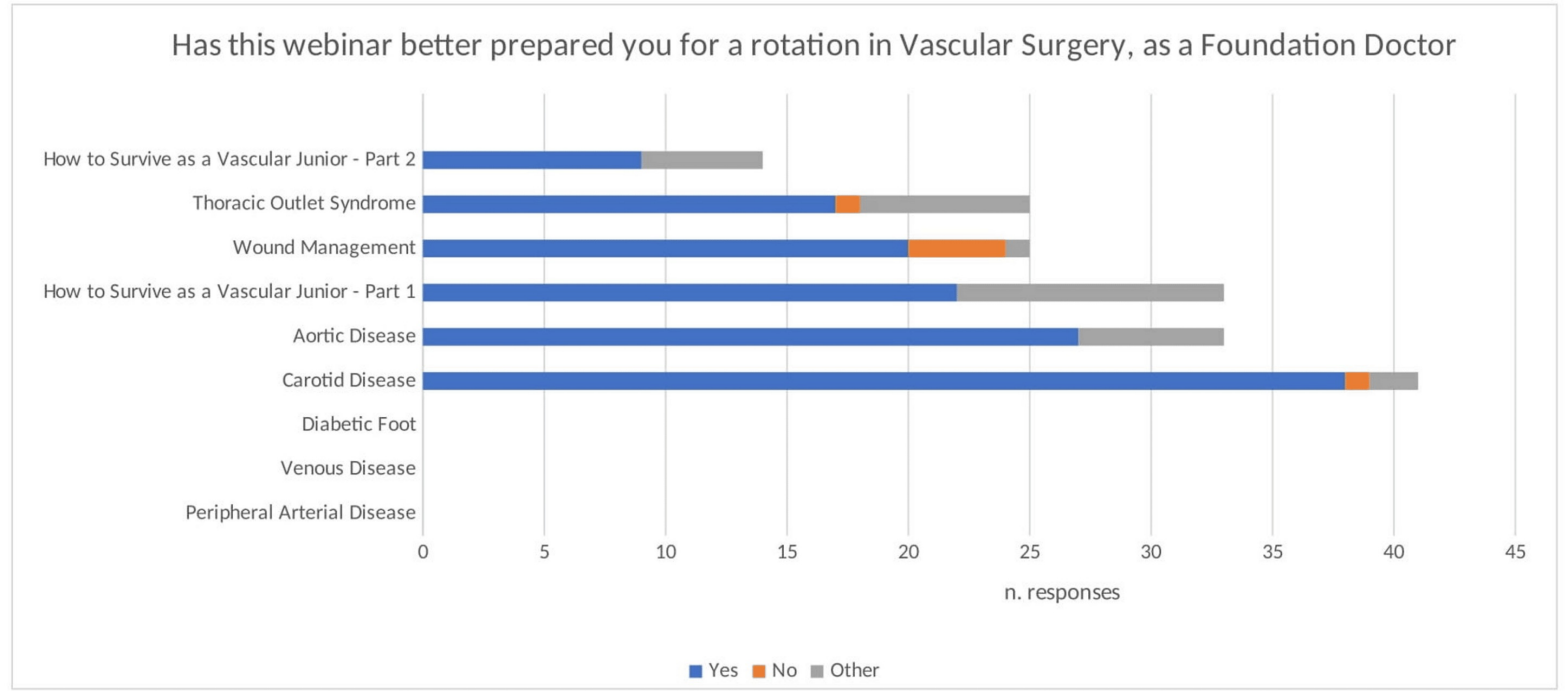
A bar chart illustrating the breakdown of feedback responses to the question ‘Do you feel this webinar has better prepared you for a job as a junior doctor in vascular surgery?’

This question was asked in feedback for six of the nine webinars. Out of the total number of respondents (n = 200) from those webinars, 133 responded yes (66.5%). The webinar on carotid artery disease had the most overwhelmingly positive response, with 38 out of the 41 participants who gave feedback responding yes (92.7%) (Figure 4). The webinar with the lowest ‘yes’ rate was wound management at 58.8% (20/34). Reasons for responding yes included an ‘increased confidence’ gained from watching the webinar, going through ‘conditions and their specific management’, and the fact the speaker was a ‘professional’ in the field. Overall, across the six webinars, six individuals responded ‘no’, with the majority being in response to the webinar on wound management (4/6, 66.7%). ‘Not applicable’ was written as an answer by eight individuals, with three individuals explaining that they were not medical students/junior doctors but instead worked as allied healthcare professionals. The remainder of the answers were equivocal and therefore could not be categorised into a binary yes/no response.

What Went Well? What Could Be Improved?

Four broad themes could be identified when reviewing feedback regarding what went well. These related to the slide show, the explanation of the topic, the presenter, and the concise length of time off the webinar. Only one participant commented regarding the professional level of the presenter, writing, “Great experienced speaker. Consultant speakers are the best.” Of note, in two webinars titled "Carotid Artery Disease" and "How to Survive as a Vascular Junior: Part 1", a quiz was used at the end. The use of multiple-choice questions was highlighted positively by participants.

In regard to feedback on what could be improved, we identified five themes. These related to the slide show, presentation skills, detail of the information, engagement with the audience, and technical issues. Most commonly, participants commented that more pictures and information would be useful on the slides. The most common feedback points related to the presenter were that the speed of presenting was too quick and that the presenter read too much information from the slides. There was a disparity in the feedback regarding the details of the information provided. Some individuals commented that more detail would have been useful, with a focus on surgical management relevant to the Membership of the Royal College of Surgeons (MRCS) examination. Others commented that the level of detail was too much for their knowledge. Across the webinars, there was a request for more clinically focused cases to explain the topics. The most frequent feedback regarding what could be improved was for more a focus on audience participation, including handouts of information, questions at the end, and quizzes. Technical issues were raised as feedback in a number of webinars; these related to difficulties viewing the slide show, difficulties with audio, and delays with the speaker setting up the presentation.

What Experience of Vascular Surgery Have You Had in Your Medical Training Thus Far?

This question was asked in the feedback for all webinars. In total, 283 participants responded (50.2%) (Table 3). This question was open-ended, and we identified four categories of responses: Zero-minimal experience, which was defined as under two weeks of experience, or zero/minimal as stated by the participant; Moderate experience, defined as greater than two weeks of experience and/or moderate experiences stated by the participant and/or description of a range of vascular conditions the participant had seen; Good experience, defined as greater than one month and/or positive experience stated by participant and/or experience in vascular surgery, including theatres, wards, and clinic settings; and Not applicable, defined as no experience or experience in vascular surgery, but the participant was not a doctor or medical student and instead was a nurse or allied healthcare professional.

**Table 3:** Experience of vascular surgery amongst participants

Participants' work xperience	N (% of total responses to question)
Zero or minimal	177 (62.5%)
Moderate	39 (13.7%)
Good	39 (13.7%)
Not applicable	28 (9.89%)

## Discussion

Our online targeted education programme was designed to provide a free, accessible resource on vascular surgery for early postgraduate doctors. The participants’ pre-session confidence levels were generally all low-rated. This result is unsurprising given that a high percentage of participants reported 0 to minimal vascular surgery experience. Our results show that across the program, there was a statistically significant increase in self-reported confidence in the topics after each session.

The high viewership for the programme resulted from our ability to advertise on social media pages and conduct sessions in the evening to maximise the number of individuals who would be free to attend. The online nature of the programme also allowed us to have speakers from a wide geographical location across the UK. Several speakers were experts in their fields. Their experience and teaching ability were reflected in the positive qualitative feedback received. There was variation in viewership per webinar seen across our targeted education programme. We believe this is related to the time spent advertising each webinar. The first webinars were advertised a minimum of one month in advance on social media platforms. The later webinars, due to delays confirming dates, were advertised for a shorter time period.

One aim of our programme was to better prepare doctors for a vascular surgery rotation in their foundation training. The results were positive in this respect, with 66% of responders agreeing with the statement and only 3% stating no, with the remainder giving equivocal answers (including that they had no planned vascular rotation or they were allied healthcare professionals). Furthermore, given the overall lack of experience in vascular surgery by our participants, we believe that our programme addresses a need for a greater education on the clinical aspects of being an early postgraduate-level doctor in vascular surgery.

Qualitative feedback identified that our webinars, which included interactive quizzes, were highlighted favourably by participants. This reflects an overall need for maintaining interactivity between the students and the teacher in the online space. The use of interactivity is described in the literature as having positive effects on learning but also on the students’ perception of social presence within an online group [17]. A 2019 meta-analysis from Pei et. al. investigated online vs. offline learning strategies for improving learning outcomes in undergraduate students [18]. The overall finding was that there were no significant differences between the two strategies concerning learning outcomes. The authors noted one study that stood out for identifying improved outcomes in the online learning cohort using delayed retention scores [19]. This study used interactive medical software, which was able to give feedback and guidance to individual students and change the difficulty of a task to fit the student’s learning needs. Maintaining interactivity in the online setting when focussing on surgical topics is plausibly more difficult, especially in the context of teaching practical surgical skills. A 2020 paper by Chick et. al. described a number of interactive online initiatives used to maintain surgical residents’ education during the COVID-19 pandemic in the United States [20]. These included online practice questions, teleconferencing, telemedicine clinics, and procedural simulation. The paper serves to describe these ventures but does not provide any data on outcomes following their introduction; however, these remain a number of important options to consider when trying to increase interactivity in online surgical resources. 

There is very limited available literature on the use of FOAMed resources for surgical education. We identified one study by Kumar et. al., which described a national six-week course covering orthopaedic joint examinations hosted on the online platform Zoom [21]. Interestingly, the authors focused on clinical examinations. A total of 92.6% of participants reported that the online videos helped to consolidate their knowledge regarding orthopaedic examinations before going straight into practice. Overall, the authors reported a statistically significant improvement in participants’ confidence and test scores after the sessions. The lack of interactivity was noted as a limitation, with students not being able to access rapid feedback on their examination technique.

A 2020 paper by Laloo et. al. described a virtual surgical education initiative for UK-based core surgical trainees (UK postgraduate doctors in their first two years of surgical training) [22]. This included 20 online lectures, which could be watched live or at a later date on YouTube. The authors reported an 89.9% satisfaction rate among participants with the series. Notably, for each session, a moderator was assigned to facilitate an interactive segment to consolidate knowledge. One of the key feedback points identified from the thematic analysis was the continued need for practical sessions. As the target audience, in this case, was surgical trainees, compared to doctors in the Foundation Programme, operative skills are more of a focus in their training. For this learning requirement, an online learning method is unlikely to yield the same outcomes as face-to-face practical teaching, such as in an operating theatre. Chao et. al., in a 2020 paper, described a virtual surgical rotation aimed at undergraduate students [23]. This included the use of first-person audiovisual technology to allow students to view surgical procedures remotely and interact with the surgeons and operating theatre staff. This method may be a viable option for integrating the need for gaining operative experience with an online platform for surgical education.

There are a number of limitations to the results described in this paper. The outcomes are limited to Kirkpatrick level one, learner reaction [24]. In order to more accurately evaluate the effectiveness of our online education programme, measuring outcomes related to Kirkpatrick levels two to four would be included. Although the participant and feedback numbers are a strength of this study, only 64.7% of the participants gave feedback, and the outcomes identified may possibly not be representative of the entire cohort. The variation in the speakers inherently will have resulted in variation in the quality and depth of the topic explored in each presentation, which will have had an effect on the outcome measures. Finally, our programme was aimed at early postgraduate doctors completing vascular surgery rotations, and these results cannot be extrapolated to claim the effectiveness of online teaching for more senior surgical doctors and medical students.

Looking forward from the MTB vascular surgery programme, there are several avenues for future development. One area is to increase the interactivity of our FOAMed resources. We are currently focussing on creating an online surgical journal club, which encourages the audience to present up-to-date research in their chosen field of interest. We also would like to focus more on basic surgical skills and are currently collaborating with surgical societies in the UK to create resources both online and for our in-person teaching conferences. Moreover, in future MTB FOAMed programmes, we are aiming to assess the long-term impact of our educational initiatives on clinical practice and, as a result, patient outcomes. A future aim is to gain a higher percentage of feedback from participants. Participants currently provide feedback in order to access their certificate of attendance. Future strategies will include reminder emails and requiring feedback to access further education sessions in a programme.

## Conclusions

The MTB vascular surgery online education programme has proven its use as an adjunct to traditional undergraduate vascular surgical education and the knowledge gained from experience in a surgical rotation as a doctor in the Foundation Programme. When comparing our programme to other online education ventures, the most striking feature is the high number of participants across our series. The programme demonstrates that an online platform can be used to provide high-quality surgical education. The programme can serve as a reference for those interested in developing FOAMed resources. 

A future consideration in online surgical education programmes is ensuring a level of interactivity with the audience, using methods such as live case discussions and surgical simulations. For programmes aimed at senior surgical trainees, a focus on more practical operative skills is needed. Furthermore, to more accurately assess the effectiveness of future programmes, the use of outcomes measuring knowledge gain and translation to clinical practice should be considered. 
